# A constant conversation: tuning into and harmonizing the needs and priorities of the body and mind

**DOI:** 10.1080/17482631.2017.1350550

**Published:** 2017-08-01

**Authors:** Annie T. Chen, Samantha J. Kaplan, Rachel Carriere

**Affiliations:** ^a^ Department of Biomedical Informatics and Medical Education, University of Washington School of Medicine, Seattle, WA, USA; ^b^ School of Information and Library Science, University of North Carolina at Chapel Hill, Chapel Hill, NC, USA; ^c^ Content Management, EBSCO Information Services, Ipswich, MA, USA

**Keywords:** Chronic illness, body awareness, body listening, self-management, sense-making

## Abstract

**Purpose:** Individuals rely upon many types of information to manage an illness, including information provided by their own bodies. This study investigated how people tune into and manage the flow of information from their bodies to manage their health. **Method:** We developed a platform for participants to share and collaboratively reflect on how they engaged in this dialogic process, in which participants contributed to a discussion on topics relating to body listening and body awareness. Though the study was open to anyone interested in or wanting to contribute to knowledge on “body listening,” the social media recruitment focused on chronic conditions requiring self-care and having overlapping symptomatology, with chronic pain as the primary characteristic. A qualitative analysis method based on grounded theory was used to analyse the data. **Results:** Six main themes emerged: learning the language, recognizing and heeding limits, experiencing emotional fatigue and despair, regulating the channel, moving from conflict to communication, and settling into an uneasy acceptance. **Conclusion:** The monitoring and filtering of information from one’s body, and the appeasement of conflicting demands and voices, is difficult work. Knowledge of this process can be used in patient education and in the development of tools to support body listening.

## Introduction

Chronic illnesses can have profound effects on people’s lives, including diminished lifeworlds, reduced incomes, withdrawal from social and recreational activities, social isolation, struggles to understand their illnesses, and threats to identity and self-esteem (Arnold et al., 2008; Crooks, 2007; Öhman, Söderberg, & Lundman, 2003; Röing & Sanner, 2015). Over the course of a chronic illness, people can learn to adapt in many ways, including becoming more attuned to their bodies, recognizing signals that the body gives, and reacting to these signals (Chen, 2016; Sångren, Reventlow, & Hetlevik, 2009). This might mean greater awareness of pain triggers and food sensitivities, becoming aware of one’s physical limits and the need to rest, and recognizing when one is going to have a hypoglycaemic episode.

Information can assist patients to learn how to manage their illness. The body can be a valuable information source, which can be combined with information from external sources to facilitate self-management (Ehrlich, St John, & Kendall, 2010). It has been argued that body awareness, defined as the ability to recognize subtle body cues, can be helpful in the management of various conditions including chronic low back pain, congestive heart failure, chronic renal failure, and irritable bowel syndrome (Mehling et al., 2009). The concept has also been characterized as a kind of awareness involving mindfulness, non-judgemental acceptance, and a sense of self grounded in experiencing physical sensations in the present (Danielsson & Rosberg, 2015; Mehling et al., 2012). In addition, patients may combine an awareness of their embodied experience with information from test results, often in the process of discerning patterns of their condition (Wolf & Veinot, 2015).

Learning how to glean information from one’s body, however, is an underdeveloped object of study. The body does not come with an instruction manual, a roadmap, or any such guide to utilizing one’s body as a source of information in the self-management of a condition or conditions. In many illnesses, patients have difficulty with the unpredictability of their symptoms (Crowe et al., 2010; Gignac et al., 2006). Uncertainty from an illness spreads into many areas of a person’s life, causing one to question the meaning ordinarily ascribed to everyday events, and dismantling a person’s view of self and reality (Mishel, 1999). After a period of disorganization, a new view of reality is formed in which uncertainty is accepted as a part of life, and the person “emerges with a more complex view of life and a more complex view of functioning” (Mishel, 1999, p. 272). Managing daily life, grappling with illness, and making sense of it become intermingled (Charmaz, 1991). Lack of available, relevant information is particularly pronounced for individuals with multiple chronic conditions (Markle, Attell, & Treiber, 2014).

The preceding literature review has shown that individuals may rely upon many types of information to manage a chronic illness, including information provided by their own bodies, but it is not always easy learning how to interpret and make use of this information. Moreover, although there is a wealth of research concerning barriers and facilitators of self-management, much less is known about the developmental patterns and sustainability of self-management over time (Audulv, 2013). In this study, we investigated how people work with information from their bodies to better manage their health. Specifically, we developed a platform for participants to share and collaboratively reflect on how they engage in this dialogic process and how they learned to do so.

## Method

### Data collection

This study was conducted on a public website that featured a discussion forum, which we called the ThinkSpace, to recognize the value of participants’ contributions. Over the course of 10 weeks, participants shared their experiences on topics relating to body listening and body awareness (Table I). Each week was introduced with an overview post that described the week’s theme, and moderators began almost every day of their week(s) with a new post inviting participants to contribute to a specific aspect of that week’s theme. Moderators’ posts were open-ended and intended to make participants feel welcome to contribute a diverse set of experiences relating to the topic. Although some moderators led multiple weeks, consecutive weeks always featured different moderators.

**Table I.** Guided Exploration schedule.

Week	Theme
1	Getting in touch with your body rhythms
2	Movement, energy and fatigue
3	Nutrition and environment
4	Pain management
5	Mood management
6	Sense-making and conveying what your body tells you in health care contexts
7	Conveying what your body tells you in life contexts
8	Tuning in to your body with arts-based techniques
9	Mindfulness as a way to get in touch with your body
10	The body as a vehicle for self-growth

Selection of topics was based on a two-step process. At the outset, the first author developed a list of seed topics based on three sets of research literature: (1) self-management of chronic illness; (2) body listening and body awareness; and (3) fibromyalgia, arthritis, multiple sclerosis, and other chronic pain and rheumatological conditions. The moderators discussed these topics, added their own, and then finalized the set of topics to explore in the Guided Exploration.

The content was collectively developed by the team through a series of meetings every two weeks. Moderators had a great deal of latitude to determine their week’s theme, their daily topics, and the content of the posts themselves. Some moderators shared their personal stories and journeys related to body listening, whereas others made questions the focus of their posts. Thus, each week had a distinct voice and character. Some weeks also featured more than one moderator, and each week had dedicated supporting moderation—moderators from other weeks participating and encouraging participants. The four moderators all had experience in the self-management of chronic conditions of interest to the study. Although moderators did not have prior experience as moderators of discussion forums, they engaged in discussions during the content development phase about what to expect once the study began. The primary investigator, who researches health-related online communities, provided background information concerning these environments, but emphasized that moderators should develop their own moderation styles.

During the weeks of the Guided Exploration, the four moderators, site administrator, and principal investigator met weekly to discuss the progress of the study. In these meetings, they would share their thoughts on that week’s developments and coordinate efforts to nurture a supportive and open environment for study participants. Altogether, the research team was comprised of 10 people, many who played overlapping roles: a project manager, faculty advisors, moderators, a site administrator, and communications and outreach personnel; and team members came from multiple disciplines, including library and information science, biomedical informatics, nursing, and public health.

Recruitment took place over university and departmental listservs; social media channels including Facebook, Reddit, and Twitter; and flyers in high-traffic areas including university campuses. The study was open to anyone over the age of 21 who was interested in or would like to contribute to knowledge on the phenomenon of “body listening.” However, our social media recruitment focused on chronic conditions requiring self-care that overlapped in symptomatology, with chronic pain as the primary characteristic. We focused on chronic conditions because of the greater need for patients’ self-management efforts and recognition of the potential benefits of online delivery methods for self-management interventions (Gogovor et al., 2017; Röing & Sanner, 2015).

### Data analysis

We employed a qualitative data analysis method based on the grounded theory method (Glaser & Strauss, 1967), which involves creating theoretical categories from data and then analysing the relationships between key categories (Charmaz, 1990). As researchers, we take a social constructionist and interpretivist perspective, acknowledging the researcher as an integral part of the research process (Brannick & Coghlan, 2007; Charmaz, 1990), in terms of our involvement in both the development of the data and the analytic process.

In analysing the data from the Body Listening Project, all posts, whether authored by participants or by moderators, were considered as data. In this study, we considered all community members to be contributing and collaborating on an equal plane. Although this study was not an ethnography, we, the authors, consider ourselves “complete members” by Adler and Adler’s (1987) triadic classification of ethnographic field roles (peripheral member, active member, and complete member), in the sense of being members of the Body Listening Project online community, as well as individuals who engaged in long-term health management and body listening.

In terms of the analytic method, at the outset, all three authors engaged in separate inductive coding, with each author developing their own set of codes. During the coding process, authors engaged in memo-writing to elucidate connections between codes, and aid in abstracting and conceptualizing themes. In grounded theory, memo-writing serves as “an interactive space for conversing … about your data, codes, ideas, and hunches” (Charmaz, 2014, p. 162). The qualitative data analysis software Dedoose was used to analyse the data.

After a period of independent coding, the first two authors engaged in weekly discussions and iterative revisions to shape the paper. This approach was influenced by Charmaz, who calls for conducting grounded theory coding in two phases, the first involving an exhaustive coding of each line or segment of data, and the second “a focused, selective phase that uses the most significant or frequent initial codes” (Charmaz, 2014, p. 113). In the selection of significant codes, concordance in the themes used by all three authors was considered a primary criterion, in addition to completeness and coherence in the description of the process of body listening. The third author reviewed the later drafts of the paper, and all three authors engaged in an iterative discussion and arrived at consensus concerning the graphical depiction of the process of body listening presented in the Discussion.

### Ethical considerations

The study protocol was reviewed and approved by the institutional review board at the University of Washington. All participants gave consent as part of the process of creating a user account to participate in the study. Participants were informed that any information that they contributed would be public, and they were encouraged not to create usernames that were similar to their real names if they did not wish their identities to be known. In the subsequent discussion of the results, we refer to all participants and moderators by their screen names.

## Results

### Sample

Over the course of the 10 week study, 234 participants registered to participate in the Body Listening Project, and altogether, participants and moderators authored 431 posts. As expected, the range of conditions reported by participants varied widely, with greater emphasis on pain conditions such as fibromyalgia and chronic pain; mental health conditions such as depression and anxiety; irritable bowel syndrome, food sensitivities, chronic fatigue syndrome, and thyroid disorders. These were all areas that the social media recruitment emphasized. Twenty-eight participants posted in the discussion forum. This participation pattern is consistent with prior research on discussion forums, in which those who participate are a fraction of the countless others that may be “lurking” (Nonnecke & Preece, 2000).

### Body listening in the context of chronic illness

In this study, we sought to answer the question of how individuals manage the flow of information from their bodies in the context of chronic conditions. Six main themes emerged: learning the language, recognizing and heeding limits, experiencing emotional fatigue and despair, regulating the channel by filtering, moving from conflict to communication, and settling into an uneasy acceptance. We now proceed to discuss each of these themes.

### Learning the language

At the outset, participants shared stories of their learned awareness to body signals and efforts to listen to these signals. For each person, the signs may be different. For example, Maud’s “siren” is a tension headache around the left eye; marygrace’s “sentinel” is a recurring hive. Participants also noticed physiological changes in their bodies due to factors such as the climate, weather, changing seasons, and geography. These include a loss of energy with the loss of sunlight (MyndiR), slowing down when the weather is cold (otmorey), and the body relaxing when it is by water (Geordie53).

Dymond observed that the body responds positively if one listens to the signals that it gives:

I took right action acting on what my body has whispered for a few years—adrenals, adrenals, adrenals … Ask the healer in my own body. So, today, ordered something for adrenal support … Each time I take right action in this way, and simply educate myself, then act, my body begins to respond in kind.

AppleStrudel shared how she got to know her body better as she explored different strategies for dealing with migraines:

I learned a lot about my body and its response to stimuli—for a long time I could not drink red wine, have any cured meats, etc. But it was precisely because I had not been listening to my body that it finally said stop and I had to re-learn how to take care of myself in order to address other less-than-healthy behaviors in my life.

Thus, learning the body’s language, its rhythms, and working with it were keys to effective health management.

### Recognizing and heeding limits

Participants engaged in extensive discussion about how they used the signals that their bodies gave them, particularly with regard to recognizing their limitations. However, they did not always respond in the same way. Although some participants endeavoured to stay within their boundaries, others were willing to go beyond them and pay for it later:

I normally pay for it over the next couple of days by being very exhausted and achy as a result. (MyndiR)

Exhaustion means I give in, I surrender. (celestewaters)

Although I know my body will need a full day to recover from even a few hours of activity I find the trade of worth it. (Boboo)

not heeding slow-down messages. (maud)

In this sense, a limit is a warning that the listener can choose to heed or to ignore. Just as some listeners erect limits to prevent their conditions from overreaching into their lives, the body erects limits to prevent itself from having to overreach into its keeper’s life. The limit’s dual nature highlights the importance of “quieting down to pay attention to what our bodies are telling us” (Gail).

### Experiencing emotional fatigue and despair

Elements of desperation and fatigue emerged throughout the study, particularly in weeks that discussed pain management. One moderator, Gail, commented, “most of us will do just about anything to relieve chronic pain”. However, relief may never come completely or at all for some, and instead they must learn to manage life with ever-present pain—almost like a night that never ends or a fever that will never break.

Resentment also emerged. In the comment below, one senses an individual quite resentful at having to play the role of mediator in the constant tug-of-war between the body’s needs and the self’s priorities:

I’m the manager of all these competing voices and have to coddle them, suppress them, have a talk with them when they get cantankerous, give them attitude adjustments and rewards. I never wanted to be a manager, no wonder they are burned out all the time! (Zepplin)

Imagine feeling as if you must negotiate with your back pain to abate for just an hour so that you can drive to the pharmacy to retrieve relief-bringing medicine, and feeling stymied in this mission by the back pain. This role is demanding and potentially unrewarding, and participants are not always prepared, but it is also unrelenting. Just as chronic pain never leaves, this conversation never quietens:

but there was always the awareness that I could be brought low and have to head to a dark room with an armful of ice packs at any moment. (Midan)

### Regulating the channel by filtering

Over the course of their experience, participants may restrict or filter the flow of information that their body sends. Participants suggested that body listening is just as much about learning what to ignore as it is about learning what to hear. Participants often framed their comments with references to time, such as “in the beginning” or “when I was first diagnosed”, and talked about how their behaviours changed, often with their condition.

One participant, Zepplin, said “I had been in pain for a long enough time that I just chose to stop listening to it, however my body could not stop reacting to it”. Underlying Zepplin’s words and the comments below is that this filtering or blunting has the potential to bring a sense of peace or is related to a sense of acceptance. Another participant, sgc1203, wrote “I wonder if we’re too obsessed with the small nuances and signs our body gives us, rather than just feel it, accept it, and let it go”. This suggests that listeners start to tune out their internal radios at some point, and that it can be a powerful choice: “I have set a limit on how my condition can affect me” (findjoyagain).

### Moving from conflict to communication

Body Listening Project community members often referred to a power struggle between the limitations and needs of the body and the desires and intentions of the members. They made comments such as “I could fool my mind but not my body” (Sami) and “our bodies will always prevent us from doing what we are not supposed to do” (AppleStrudel), indicating that at times it felt as if their bodies knew more than they did. While this can contribute to feeling as if the body is the dominant partner in the relationship between body and self, there was also acceptance of the body’s signals and the choice to listen:

At the end of the day, though, my body will tell me what it needs and what it wants less of. It’s up to me to pay attention and obey. (AppleStrudel)

If the body knows better, it could be said that the mind forgets better. The mind will try to look past, ignore, repress, or any number of actions that will allow it to surpass an unwanted physical or emotional sensation, but the body cannot do the same:

Sometimes we have physical manifestations of stress, such as muscle tension, that give us clues about our moods when we may not be otherwise aware. (marygrace)

Participants learn to negotiate between the different communication styles of mind and body as they handle their everyday life experiences. While to some it appears as a power struggle, it can also be viewed as a constant conversation between sparring partners. Instead of two entities fighting for dominance, at least one knows that they need to cooperate to compromise. Our participants were not so much at the mercy of their conditions; rather, they were trying to mediate the occasionally competing priorities of mind and body.

Thus, if we consider the body as an ever-changing information source communicating its own priorities, limitations, and needs, this power struggle begins to resemble a conversation rather than a conflict. Body listening, while difficult at times, appears to empower participants and allow them to regain a semblance of control or achieve a sense of peace in regard to their conditions:

My body is telling me, not now, the suggested dental work needs to wait, you can’t cope with the stress of a root canal on a tooth that isn’t hurting you right now… My body wants a healer. I am open to facilitation, I am open as well to being my own healer, the right information and support comes to me. (dymond)

Body listening is part of the illness journey and allows for an important separation from one’s condition, or conditions, so that the individuals and their lives are not defined by their suffering.

### Settling into an uneasy acceptance

Acceptance of one’s own health situation was often spoken of. Acceptance is a state that involves being okay with one’s health situation and the aspects of it that are outside one’s own control: “while everything in me and around me may never settle down, understanding my body rhythm and inner life for what they are, truly can help me get to the best place of management” (dymond). This acceptance can involve a conscious choice:

“I feel like the obstacle can be the path itself. I didn’t choose the path however choosing to resent treading it contributes to diminished health” (Zepplin).

Participants seemed to accept that their conditions were potentially permanent but seemed ill at ease with the potentially permanent changes to their lived experience. There were reflections upon members’ current states, including a recognition of the impermanence of one’s state of being:

I feel like I have grown in my journey, and I feel proud of myself for being in that place. But there are other times when I feel “I am not going out like that!” and rail against what is happening to me. I go back and forth on accepting my illnesses. (Gail)

Working to live in the present, to accept myself as I am, and to loosen the hold of my vision of how my body should feel and what my life should be like. This is really all about managing expectations. (Rachel)

## Discussion

### Body listening in everyday life

**Figure 1.**
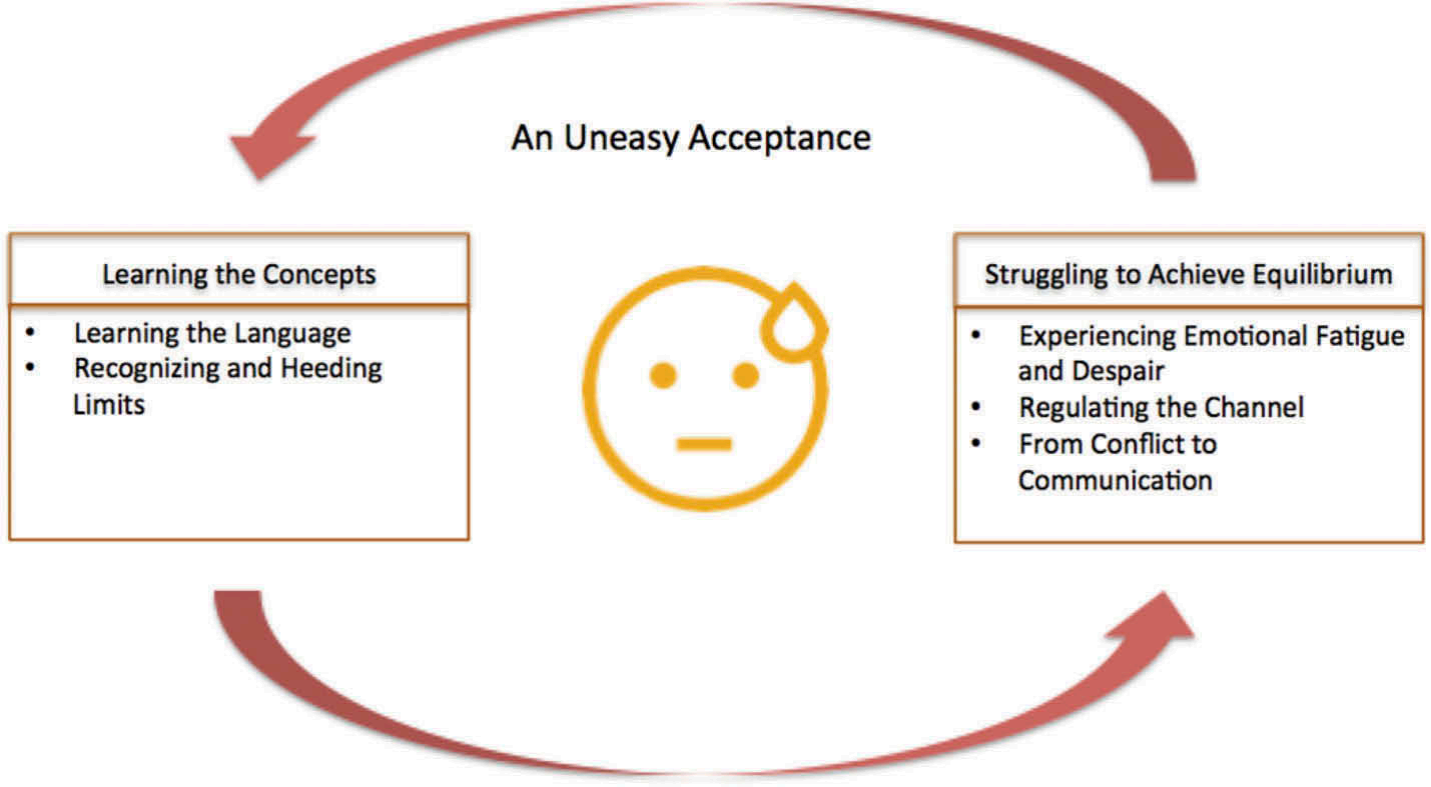
Body listening in everyday life.

We now consider how the themes that emerged in this study illustrate the everyday experience of body listening. At the outset, learning the language and recognizing and heeding limits might be considered foundational concepts that people learn as they tune into their bodies (Figure 1). The experience of body listening, while potentially of great benefit to the individual, can also be extremely trying. People constantly experience resistance in the form of emotional fatigue and despair, and they learn to regulate the channel by filtering and harmonizing conflicting voices to achieve communication.

In Figure 1, arrows go back and forth between “Learning the Concepts” and “Struggling to Achieve Equilibrium” because this is a constant, dialogic process. The process of learning does not stop, as one continues to learn new signals as a result of new information exposure, and as one experiences physiological changes over time. And although the mind learns the concepts, one generally continues to experience resistance and moderate the resulting tensions. Thus, the acceptance is an uneasy one, and there continue to be ups and downs in the experience of body listening and, more broadly, health management. This entire process is grounded and self-contained within an individual’s reality. Others can help, but they can never fully do for the person what the person can do for himself or herself.

### Reflections on the mind–body dualism

A sense of the mind and body at odds emerged in this study, and is also a recurrent theme in the literature. For example, research has reported externalization/objectification of the body relative to the self (Crowe et al., 2010) and characterization of the body as “treacherous” (Råheim & Håland, 2006). It has also been observed that body awareness can be either positively or negatively toned, and that it can be helpful to encourage patients to shift attention from negatively toned body awareness to activity in the outside world (Lööf, Johansson, Henriksson, Lindblad, & Bullington, 2014).

In the medical literature, there has traditionally been concern that heightened body awareness can lead to somatosensory amplification and is maladaptive for clinical outcomes, but body awareness, when considered as the ability to recognize subtle body cues, can be helpful for the management of chronic conditions, and transcendence of the dualism to achieve mind–body integration is seen as an ultimate goal of body awareness therapies (Mehling et al., 2011). Moreover, it has been argued that dualistic notions of the body as object and the mind as subject can devalue experiences that are necessary for healing, and that a reconceptualization of the body as a partner and informant can enhance sensitivity, listening and creative coaching in nursing care (Wilde, 2003).

This study also produced examples of the mind–body dualism, frustration dealing with the body, and conscious efforts to selectively tune out those voices. However, we also witnessed efforts to work with the body, including trying to “follow its desires” (celestewaters), to ask it what it wants to eat (Rachel), and to appreciate and understand it. Thus, there was also an effort to work with the rhythms of the body, and it may be beneficial to consider this conceptualization.

### Patient education for body listening

This study raises the question of how patient education might help people to learn to become more adept at managing the flow of information from their bodies. Our participants voiced emotional fatigue and occasional despair, particularly as it related to the onerous task of body listening and conveying what is learned to others. Ancker et al. (2015a) also reported that healthcare providers and patients with multiple chronic conditions found managing information a form of invisible labour—much like body listening. Thus, the question arises of how we can support people as they engage in these tasks, or in the learning of these tasks.

Patient education programmes such as body awareness therapy, which employ movements, breathing, massage, and awareness to try to restore balance, freedom, and unity of body and mind (Gard, 2005), have been explored for a variety of conditions including fibromyalgia, chronic pain, irritable bowel syndrome, and depression (Danielsson & Rosberg, 2015; Eriksson, Möller, Söderberg, Eriksson, & Kurlberg, 2007; Gard, 2005). With regard to diabetes, there are efforts to develop patient education programmes aimed at increasing patients’ self-awareness, specifically through recognition of the symptoms that signify hypoglycaemia or hyperglycaemia for them (Hernandez, Bradish, Rodger, & Rybansky, 1999; Hernandez, Hume, & Rodger, 2008).

The findings of this study suggest areas that healthcare providers or peer educators could emphasize in working with people with chronic conditions. Specifically, they could help patients to consider what types of signs to look for, explore with patients the right amount of body listening to do to improve or manage their health without it becoming an onerous task, and counsel them to reduce experiences of information overload and powerlessness. This aligns with a study of rheumatoid arthritis patients, which reported that patients struggled to balance their ideals with reality, and suggested that providers work with patients to develop the right balance of lifestyle habits (Malm et al., 2016).

### Tools to support body listening and sense-making about bodily sensations

For many of our participants, their bodies are an information source that they interact with, study, interpret, negotiate with, and engage. When learning to listen or first beginning to listen, participants monitored and perhaps listened too much—exhibiting monitoring behaviour and experiencing information overload. Over time, they became more astute about what information was actually relevant or useful to them and learned to filter out other information. The experience sounds similar to joining a social network, such as Twitter, and immediately needing to learn a new information environment’s norms and language. Once joined, perhaps the novice follows or friends a lot of other users with varying viewpoints and then, over time, whittles them down to a trusted or preferred set.

However, unfollowing someone is infinitely easier than learning to ignore a recurrent pain or muting a bodily signal. As many mHealth devices and applications move towards constant monitoring and generating of bodily metrics, this raises important questions about developing support for muting the body’s signals rather than amplifying them. Helping individuals to learn how to selectively apply blunting is an area of opportunity for researchers and health practitioners.

Findings from this study also reaffirm the need for appreciation of the role of sense-making and emotions in chronic illness. Listening to the body and managing it is difficult work that can result in emotional fatigue and despair. Participants develop their own ways of managing this work through dialogue with their bodies, monitoring, and filtering of information that their bodies provide, but the development of these skills takes time, and the constant and iterative process demands a great deal from people who are already suffering and could benefit from additional support.

As Sanches et al. (2010) has argued, there may be ways that we can reduce this burden and even encourage reflection. Some community members, such as dymond, spoke of journaling off and on. But other members, like the participants in Ancker et al. (2015a) study, did not have “unlimited enthusiasm for tracking their own health data”. One moderator, Rachel, noted, “I kept a small log of my pain for about a year and then gave up, which I’m embarrassed to admit because I know how beneficial it can be”. Despite knowledge of the perceived benefits, Rachel did not consider the practice of self-monitoring worthwhile. It is possible that the benefits are exaggerated or not true, but also that even if the benefits are real, they place too heavy a burden on the person.

In developing technological interventions to aid body monitoring, we must consider whether they add to or ameliorate the weight that individuals feel. The perceived benefits may not be enough, recalling the age-old question: is ignorance truly bliss? Are participants’ lives improved by listening to their body?

### Methodological considerations

The project differs from much of the extant research based on patient experience in various ways. First, this project informed people from the outset that the experiences they shared would be considered data, and the entire resource was made publicly available. We chose this data-collection design because there are many who read user-generated content but do not post themselves, typically known as lurkers (Sun, Rau, & Ma, 2014), who may benefit from the content. There was also the potential to include more voices than one might be able to engage with in a focus group or intervention study. On a topic such as “body listening”, where the definition itself is open to interpretation, the attempt to maximize the diversity of interpretation was particularly invaluable.

The ThinkSpace allowed for participants to respond to moderators and to one another, and encouraged them to build on the experiences of others. In addition, participants benefited directly from interacting with one another, as shown by the exchange below:

Wow!! Whoa! I feel that MyndiR you have written the holy grail synopsis of my life biorhythm! … I’ve classified myself “low energy” for decades to try to explain these feelings instead of respecting the very rhythm that is mine … I do feel the combination of factors on my levels of productivity and such, as well as external and internal temperatures, but I don’t think I ever accepted my rhythm for what it is and narrowed it down like this. (QnVz)

While every person is unique, there are clear common pathways and experiences in body listening. Regardless of what compels a person to listen to their body, at some point they are likely to experience fatigue and despair from listening—could this be mitigated with a warning that these are common experiences for novice body listeners? Instead of feeling further isolation, this could engender a sense of community membership and empowerment. This approach has also been advocated in previous literature (Lidén, Bjork-Bramberg, & Svensson, 2015).

The findings of this study have limitations. This project employed a data-collection instrument that was more conducive to individuals who were fluent in digital settings, comfortable sharing their experiences online, and able to articulate their experiences in a written medium. In addition, to identify a set of topics that could be covered within the 10 week study period, a particular set of chronic conditions was used as the basis for the seed topics. Thus, the topics might not have resonated with all of the participants in the study. Lastly, this study employed data that was collaboratively produced by a limited number of people, in a finite amount of time. There are areas in which we believe that the themes that emerged could potentially be richer or more detailed with a larger sample. In the future, research conducted on body listening using such a collaborative platform could focus on other populations, and potentially augment the conceptualization presented in this article.

## Conclusions

In this study, we investigated how people tune into and manage the flow of information from their bodies to better manage their health. Although listening to their body can help a person to manage their health, the task of body listening can be a trying one. There is much to be done in the monitoring and filtering of information, and in the appeasement of conflicting demands and voices. However, with time, this activity can become more of a conversation than a conflict. Knowledge of how this process occurs can be used to educate people about how to tune into their body’s signals and to develop tools to support body listening.

While we have learned much about how individuals experience body listening, and how it influences their lived experience, there is still much that we do not know about how people learn to do it. This is an area that deserves further examination. Additional future directions could explore their ability to teach and learn from one another. The act of body listening allows for the conceptualization of bodily experiences as interrelated events and signals that should not be silenced or muted; rather, they should be heard and even, perhaps, given responses.

Those who listen to their bodies are themselves the information and seeker, their own divining rod and diviner, a unique language with only one speaker and one learner/interpreter. This relationship is easily mired by conflicts of interest and ego, unreliable or inconsistent communication from body to self, and interpretation error, among many other potential pitfalls. However, there is no other equivalent source that can tell a person what their body can. If an individual can effectively learn their body’s language, they may be able to manage their conditions more effectively, potentially improving their quality of life.
